# Effects of pre-meal dashi consumption on food preferences and sweet taste sensitivity in patients with type 2 diabetes: a pilot randomized controlled trial

**DOI:** 10.3389/fnut.2026.1903008

**Published:** 2026-10-28

**Authors:** Akiko N. Beppu, Kento Mitsushio, Megu Y. Baden, Takuya Kagisaki, Sarasa Kato, Tomomi Horii, Chisaki Ishibashi, Yoshiya Hosokawa, Yuya Fujishima, Junji Kozawa, Hitoshi Nishizawa, Yusuke Furuya, Akio Yonekura, Takatoshi Yamashita, Taro Kawabe, Iichiro Shimomura

**Affiliations:** 1 Department of Metabolic Medicine, Graduate School of Medicine, The University of Osaka, Suita, Japan; 2 Department of Lifestyle Medicine, Graduate School of Medicine, The University of Osaka, Suita, Japan; 3 Department of Diabetes Care Medicine, Graduate School of Medicine, The University of Osaka, Suita, Japan; 4 Department of Metabolism and Atherosclerosis, Graduate School of Medicine, The University of Osaka, Suita, Japan; 5 Cancerscan, Inc, Tokyo, Japan; 6 Kubara Honke Group Co., Ltd, Fukuoka, Japan

**Keywords:** dashi, food preferences, sweet taste sensitivity, type 2 diabetes, umami

## Abstract

**Background & Aims:**

Impaired sweet taste sensitivity and preferences for energy-dense foods have been reported in patients with type 2 diabetes, although practical strategies to modify these preferences remain limited. We investigated whether consuming dashi, a soup stock rich in umami, before meals could alter food preferences and sweet taste sensitivity in patients with type 2 diabetes.

**Methods:**

We conducted an open-label randomized controlled trial with an initial 4-week parallel-group period followed by an exploratory 4-week period in which participants received the alternate intervention without a washout interval. Twenty patients with type 2 diabetes were randomly assigned to consume either dashi (dashi group) or hot water (water group) before breakfast and dinner during the initial phase. The primary outcome was the between-group difference in the change in sweet taste threshold from baseline to week 4. Secondary outcomes included the feasibility of pre-meal dashi intake, food preferences, biomarkers, anthropometric parameters, and dietary intake. Second-period outcomes were summarized descriptively.

**Results:**

Eighteen participants (90%) completed the week 4 assessment, and adherence to the dashi intervention was 97%. The adjusted between-group difference in sweet taste threshold was −5.2 g/L (95% CI, −13.1 to 2.9; *p* = 0.19). In exploratory within-group analyses, preference for non-sweet fatty foods and sweet carbohydrate foods decreased in the dashi group; however, the corresponding adjusted between-group differences were −11 points (95% CI, −28 to 7; *p* = 0.22) and −8 points (95% CI, −22 to 7; *p* = 0.29), respectively. Among participants with impaired sweet taste sensitivity at baseline, 3 of 5 (60%) in the dashi group and 0 of 4 (0%) in the water group improved to normal sensitivity (*p* = 0.17).

**Conclusions:**

In this pilot randomized controlled trial, pre-meal dashi intake was feasible, but superiority over hot water was not demonstrated. Exploratory findings on food preferences and sweet taste sensitivity are hypothesis-generating and warrant confirmation in further large-scale, independently conducted trials.

**Trial registration:**

This study was registered with the University Hospital Medical Information Network (UMIN000050054).

## 1 Introduction

The global increase in the number of patients with type 2 diabetes has led to an urgent need for more effective treatment strategies, particularly dietary modification, which serves as the foundation of diabetes treatment. Consequently, the development of effective dietary interventions for patients with type 2 diabetes has become a critical research focus.

“Internal food cues”, such as hunger, food cravings, and emotional eating, have been demonstrated to act as barriers to adherence to dietary interventions (1). Previous studies have shown that individuals with obesity tend to prefer energy-dense foods, including savory high-fat foods and sweet foods (2), and excessive intake of sweets and processed meats has been associated with poor glycemic control in patients with type 2 diabetes (3). In addition, patients with type 2 diabetes often exhibit lower sweet taste sensitivity than individuals without diabetes (4–6). Among people with impaired glucose tolerance, reduced sweet taste sensitivity has been associated with higher sugar intake and vascular complications (7). Taken together, these findings suggest that altered taste perception and unhealthy food preference may be clinically relevant barriers to effective dietary modification in type 2 diabetes. Although food preference is clinically relevant to dietary adherence, nutrient-based and culturally adapted tools to assess food preferences in Japanese populations have been limited. Recently, the Japan Food Preference Questionnaire (JFPQ), which consists of foods commonly consumed in Japan, was developed and shown to capture food preference patterns associated with actual dietary intake, particularly for fat and carbohydrate preferences (8). These findings suggest that the JFPQ may provide a useful framework for evaluating intervention-related changes in food preferences in Japanese populations.

Behavioral nutrition interventions, including dietary counseling, mindful eating, and strategies to improve adherence to healthy eating patterns, are fundamental components of diabetes management (9). However, long-term adherence to these interventions remains challenging (9), partly because food preferences and taste perception influence food choices (10). Therefore, interventions targeting sensory perception and food preference may complement conventional dietary approaches. Dashi, a fundamental component of traditional Japanese cuisine, is typically prepared using ingredients such as dried fish (e.g., bonito flakes, dried sardines, and flying fish), kelp, and dried shiitake mushrooms. It is rich in umami compounds including glutamate, inosinate, and guanylate, which act synergistically to enhance umami perception (11). Umami has been reported to increase food palatability and satisfaction (12, 13), and previous experimental studies suggest that monosodium glutamate (MSG) can enhance satiety (14) and reduce subsequent intake of energy-dense or savory foods (15, 16). MSG supplementation has also been associated with lower sugar intake (17), higher GLP-1 concentrations (18), modulation of appetite and blood glucose levels (19), and altered taste receptor gene expression (17, 20). These observations suggest that umami exposure could influence both taste sensitivity and food preference. However, previous studies have mainly focused on single umami compounds, such as MSG, and to our knowledge, no studies have examined whether a practical pre-meal dashi intervention can modify food preferences in adults with type 2 diabetes.

Because dashi provides synergistic umami stimulation and can be easily incorporated into daily meals, it may represent a culturally acceptable strategy to support dietary behavior change. We therefore conducted a pilot randomized controlled trial to evaluate the feasibility of pre-meal dashi consumption and whether it modulates food preferences and sweet taste sensitivity in patients with type 2 diabetes.

## 2 Methods

### 2.1 . Study design and participants

Participants were adults with type 2 diabetes who were receiving treatment at the Department of Metabolic Medicine, Osaka University Hospital, between 2023 and 2024 and who expressed interest in participating in the study. Exclusion criteria included diseases that reduce saliva flow (e.g., Sjögren syndrome); use of drugs that cause taste disorders (e.g., anticancer drugs); diseases associated with zinc deficiency (e.g., dialysis, liver cirrhosis, inflammatory bowel disease); severe liver or kidney dysfunction; thyroid disorders; and allergies to blue-backed fish, shiitake mushrooms, or wheat. This was an open-label, randomized, two-period intervention study. The primary comparison was prespecified and based on the first 4-week parallel-group period. Following the primary assessment at week 4, participants received the alternate intervention for a further 4 weeks without a washout interval. Because carryover and period effects could not be excluded during the second period, formal between-group comparisons were restricted to the first period, whereas second-period data were summarized descriptively. The decision to summarize second-period data descriptively was not prespecified. After providing written informed consent, patients were randomly assigned to two groups using a stratified permuted block method, with age, sex, and BMI as allocation factors. The randomization sequence was generated by the co-first author responsible for the REDCap randomization system. Participants were enrolled by the study investigators at Osaka University Hospital. For each participant, after written informed consent was obtained and enrollment was completed, the enrolling investigator accessed the REDCap randomization module, and the group assignment was revealed only at that time. Due to the open-label design of the study, neither participants nor outcome assessors were blinded to group assignment, including those evaluating taste sensitivity. Laboratory personnel responsible for biochemical measurements were not provided with information regarding group assignment. Previous intervention studies evaluating umami taste have typically included 15–30 participants per group (17, 19). The sample size for the present study was initially determined based on the following assumptions: the mean change in sweet taste threshold was 1.0 in the dashi intervention group and 0.0 in the control group, with a common standard deviation of 1.0. Using a two-sided significance level of 5% and 80% power, the required sample size was calculated to be 17 participants per group. Assuming a 15% dropout rate, the target sample size was set at 20 participants per group (40 participants in total). However, recruitment was slower than anticipated, and only 20 participants were randomized during the study period, below the planned target of 40. The decision to stop recruitment was made before examination of outcome data. Accordingly, the present report should be interpreted as a pilot trial and all efficacy analyses as exploratory.

### 2.2 Intervention

The primary randomized intervention period was set at 4 weeks based on previous studies investigating the effects of MSG on food preference (16). During the intervention period, participants consumed either dashi dissolved in hot water before breakfast and dinner (dashi group) or hot water alone (water group). The dashi consisted of 2.5 g of powdered dashi (Shiageno Kona Dashi, Kayanoya, Kubara Honke Group Co., Ltd. Japan) dissolved in approximately 50 mL of hot water. The dosage of 2.5 g per serving was selected with reference to previous studies on MSG administration (17), taking into account the synergistic effects of glutamic acid, inosinic acid, and guanylic acid. Each 2.5 g serving provided 0.3 g protein, 0.01 g fat, 1.74 g carbohydrate, 0.15 g salt equivalent, 22.2 mg glutamic acid, 61.5 mg inosinic acid, and 49.8 mg guanylic acid. These composition values were based on measurements performed by the manufacturer for this study. According to the product ingredient declaration, the ingredients were kelp extract powder, kelp powder, dried sardine extract powder, shiitake mushroom extract powder, shiitake mushroom powder, starch hydrolysate, and yeast extract. Participants in the water group consumed an equivalent volume (50 mL) of hot water before breakfast and dinner during the same period. Adherence to the intervention was assessed using participant self-recorded logs and cross-checked against the remaining amount of dashi returned at follow-up visits.

### 2.3 Outcome assessments

The prespecified primary outcome was the between-group difference in the change in sweet taste threshold for sucrose from baseline to 4 weeks. Secondary outcomes included adherence and completion rate, changes in food preferences, changes in salty and umami taste thresholds, biomarkers, anthropometric measurements, and dietary intake assessed by the Food Frequency Questionnaire (FFQ). Within-group pre-post changes and changes in taste sensitivity categories according to baseline sensitivity were considered exploratory analyses.

Food preferences were assessed using the Japan Food Preference Questionnaire (JFPQ), which we have recently developed (8). Briefly, foods were classified into carbohydrate, fat, or protein groups based on their ratios of carbohydrate, fat, or protein to total energy. Foods with a carbohydrate ratio of ≥70 % were categorized as carbohydrate group, those with a fat ratio of ≥40 % as fat group, and those with a protein ratio of ≥30 % as protein group. In addition, foods with a high dietary fiber content (≥1 g per 100 g of edible portion) and low energy (< 100 kcal per 100 g of edible portion) were selected for the dietary fiber group. For the carbohydrate and fat groups, each food was further divided into two subgroups: a sweet subgroup with more than 10 g of total sugars (including monosaccharides and disaccharides) per 100 g of edible portion and a non-sweet subgroup with less than 10 g of total sugars. Preference for each food item was rated on a visual analog scale from 0 (not at all) to 10 (extremely), with pictures of the foods displayed to allow for associations through visualization. Total scores were calculated for the carbohydrate, fat, protein, and dietary fiber groups. Because the maximum score differed for each nutrient group, we calculated the percentage of the total score to the full score for each nutrient group. In the original development study, JFPQ scores were positively correlated with corresponding dietary intake estimated using an FFQ across all four nutrient groups in the overall sample, providing preliminary support for construct validity (8). A subsequent longitudinal study reported decreases in JFPQ preference scores for carbohydrates and fat at 3 and 6 months after semaglutide initiation, suggesting potential sensitivity to change (21), although formal test–retest reliability, responsiveness, and a minimal clinically important difference have not yet been established.

Taste sensitivity for sweet, salty, and umami flavors was assessed using the whole-mouth gustatory test of sucrose, sodium chloride and MSG solutions (Supplementary Table 1) (22). The whole-mouth gustatory test has previously demonstrated good test–retest reproducibility in healthy adults (23). Before study initiation, the investigators conducted practice sessions to standardize the testing procedures. All taste sensitivity assessments were conducted by trained investigators according to standardized procedures. For each participant, the baseline and week-4 taste assessments were generally performed by the same investigator, who was aware of group assignment. Participants rinsed their mouths with distilled water and held approximately 15 mL of each lowest-concentration solution in the oral cavity for around 5 seconds. If the taste was not correctly identified, the next higher concentration of the solution was applied. Solutions were presented in ascending order of concentration, and the lowest concentration at which the taste was correctly identified was defined as the taste threshold; lower threshold values indicated greater taste sensitivity. Participants were categorized into two groups based on their baseline taste sensitivity: normal sensitivity and reduced sensitivity. The cut-off thresholds were 10 g/L for sweet, 0.6 g/L for salty, and 0.8 g/L for umami, and participants who failed to recognize these concentrations were classified as having reduced sensitivity (22).

Biomarkers and body composition were assessed at baseline and at the follow-up visits through physical examinations and blood tests. Body weight, body fat mass, and lean mass were measured using a body composition analyzer (MC-780A-N, TANITA Co. Ltd, Tokyo, Japan). The participants' dietary intake before and during the study was evaluated using the FFQ (Educational Software Co., Ltd., Tokyo, Japan). Physical examinations, blood sampling, and the JFPQ were conducted in the fasting state.

### 2.4 Statistical analysis

Continuous variables are presented as mean (SD), and categorical variables are presented as number (%). For descriptive comparisons of baseline characteristics, continuous variables were compared between groups using Welch's *t*-test and categorical variables using Fisher's exact test. Analyses were conducted on a complete-case basis for each outcome, including participants with both baseline and 4-week data for the relevant outcome. These analyses were not intention-to-treat analyses. Two participants in the dashi group did not complete the week-4 assessment and were therefore excluded from the corresponding analyses. For the primary outcome, one additional participant in the water group was excluded because an incorrect taste identification during the baseline measurement prevented determination of the threshold. Missing outcomes were not imputed because model-based imputation would have been highly assumption-dependent in this small dataset. For the same reason, an imputation-based sensitivity analysis was not performed. Between-group differences in continuous outcomes at 4 weeks were evaluated using analysis of covariance (ANCOVA), with the 4-week value as the dependent variable, intervention group as the independent variable, and the corresponding baseline value as a covariate. Adjusted between-group differences with 95% confidence intervals (CIs) and *p*-values were reported. The normality of within-participant changes from baseline to week 4 was assessed using the Shapiro–Wilk test. Exploratory within-group changes were analyzed using paired *t*-tests when the paired differences were approximately normally distributed and using Wilcoxon signed-rank tests otherwise. For taste sensitivity categories, proportions were compared between groups using Fisher's exact test. Statistical analyses were performed using R software (version 4.2.2, R Foundation for Statistical Computing, Vienna, Austria). All statistical tests were two-sided. For the prespecified primary outcome, a *p*-value < 0.05 was considered statistically significant. Given the smaller-than-planned sample size and the exploratory nature of this pilot study, *p*-values for secondary or exploratory outcomes are reported as unadjusted nominal *p*-values.

## 3 Results

### 3.1 . Participant characteristics

The participant flow chart is shown in Figure 1. A total of 20 patients with type 2 diabetes were enrolled and randomly assigned to either group (10 participants per group). One participant in the dashi group transferred to another hospital, and another did not attend the week-4 assessment and was lost to follow-up. Eighteen patients (8 in the dashi group and 10 in the water group) completed the 4-week primary assessment (90% overall completion of the primary period). Adherence to the dashi intervention during the primary period was 97%. Baseline clinical characteristics of the participants are shown in Table 1. The mean age was approximately 69 years, there were almost equal numbers of men and women, and the mean duration of diabetes was approximately 20 years. No adverse events occurred during the 8-week study period.

**Figure 1:**
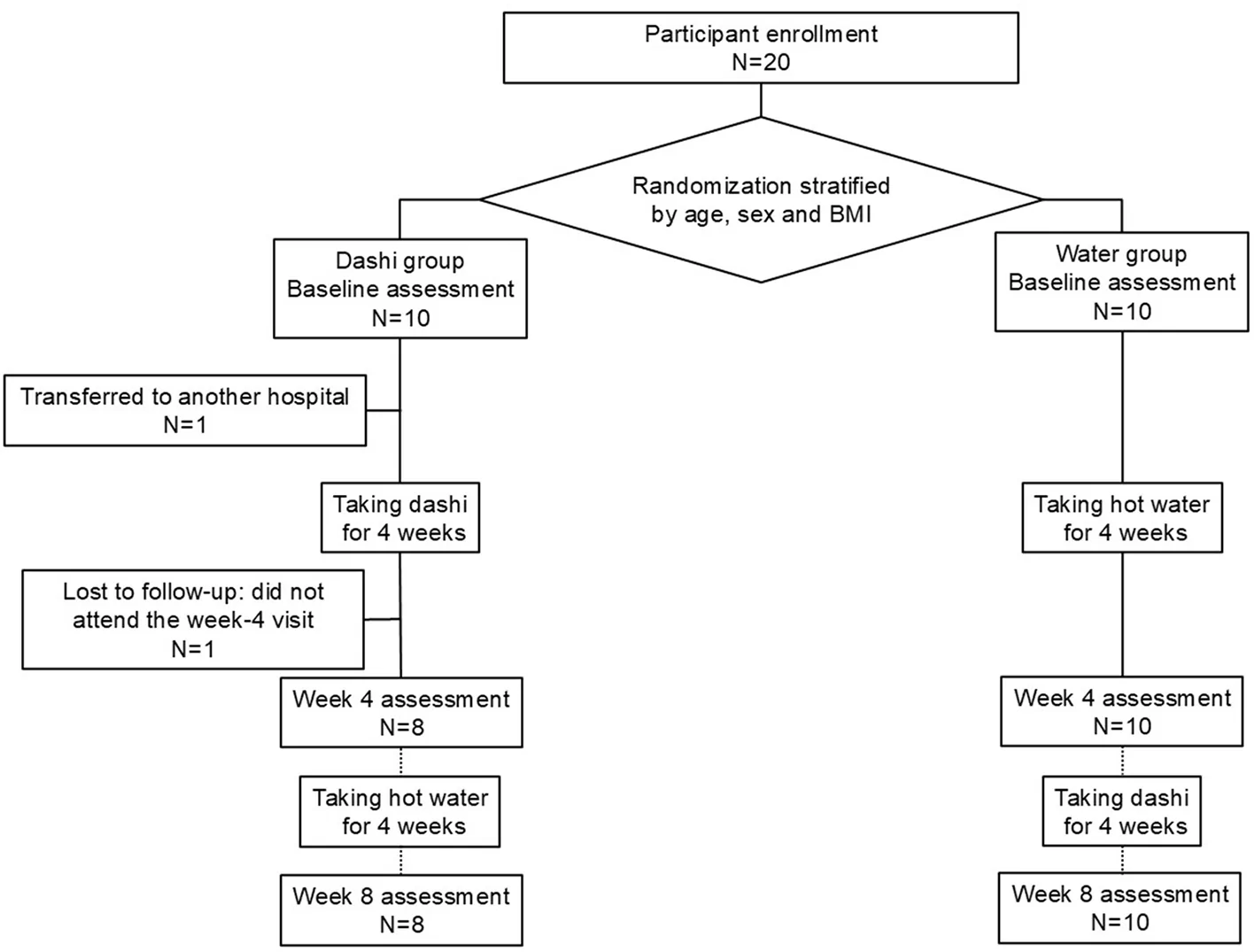
Participants flow through the study. Participants were randomized 1:1, with stratification by age, sex, and BMI. Solid lines indicate the initial 4-week randomized parallel-group period used for the primary comparison. Dashed lines indicate the subsequent 4-week period, during which participants received the alternate intervention without a washout interval. Because carryover and period effects could not be excluded during the second period, second-period outcomes were summarized descriptively.

**Table 1:** Clinical characteristics of the participants.

Characteristics	Dashi group (*n* = 8)	Water group (*n* = 10)	*p-*value
Age (years)	68.8 ± 14.4	69.6 ± 16.2	0.91
Sex (male/female)	4 (50)/4 (50)	6 (60)/4 (40)	1.00
BMI (kg/m^2^)	25.5 ± 3.2	24.8 ± 2.6	0.63
Duration of diabetes (years)	21.0 ± 6.6	16.7 ± 10.9	0.32
TG (mmol/L)	1.51 ± 0.83	1.01 ± 0.27	0.14
LDL-C (mmol/L)	2.67 ± 0.77	2.52 ± 0.51	0.65
HbA1c (NGSP, %)	7.6 ± 0.7	7.1 ± 0.8	0.21
eGFR (ml/min/1.73 m^2^)	70 ± 34	70 ± 29	0.98
AST (U/L)	18 ± 4	18 ± 4	0.76
ALT (U/L)	15 ± 5	18 ± 8	0.44
Sweet taste threshold (g/L)	30.0 ± 38.1	25.6 ± 24.0	0.78
Salt taste threshold (g/L)	2.0 ± 1.5	1.1 ± 0.8	0.18
Umami taste threshold (g/L)	2.3 ± 2.0	2.7 ± 3.7	0.80

Values are presented as mean ± SD or number (%). Baseline characteristics are presented for participants who completed the initial 4-week assessment. Sweet taste threshold data were available for nine participants in the water group. Baseline *p*-values are provided for descriptive purposes only and should not be interpreted as evidence of the success or failure of randomization. BMI, Body Mass Index; NGSP, National Glycohemoglobin Standardization Program; TG, triglyceride; LDL-C, low-density lipoprotein cholesterol; AST, aspartate aminotransferase; ALT, alanine aminotransferase.

### 3.2 Primary outcome

The primary outcome, change in sweet taste threshold after the 4-week intervention, is presented in Table 2. The adjusted between-group difference in sweet taste threshold was −5.2 g/L (95% CI, −13.1 to 2.9; *p* = 0.19). In exploratory analyses, changes in sweet taste sensitivity stratified by baseline sweet taste sensitivity (normal vs. reduced) are presented in Table 3. Among participants with reduced sweet taste sensitivity at baseline, 3 of 5 (60%) in the dashi group improved to normal sensitivity, compared with 0 of 4 (0%) in the water group (*p* = 0.17). Given the small numbers, this exploratory finding should be interpreted cautiously.

**Table 2:** Change in sweet taste threshold from baseline to 4 weeks.

Measures	Dashi group (*n* = 8)	Water group (*n* = 9)
Baseline	30.0 ± 38.1	25.6 ± 24.0
4 weeks later	13.1 ±5.9	17.8 ±9.7
Change from baseline (95% CI)	−16.9 (−46.8, 13.0)	−7.8 (−24.0, 8.4)
Within-group *p* value	0.20	0.42
Adjusted between-group difference (95% CI)	−5.2 (−13.1, 2.9)	
Adjusted between-group *p* value	0.19	

Data are presented as mean ± standard deviation. Taste threshold values are expressed in g/L. Changes were calculated as the difference between baseline and 4 weeks after intervention. Within-group analyses are exploratory and presented for descriptive purposes. Adjusted between-group difference, corresponding 95% CI, and *p*-value were estimated using ANCOVA. Within-group *p*-values were calculated using the Wilcoxon signed-rank test. CI, confidence interval.

**Table 3:** Changes in sweet taste sensitivity according to baseline sensitivity.

Baseline	Outcome	Dashi group (*n* = 8)	Water group (*n* = 9)	*P* value
Reduced		*n* = 5	*n* = 4	0.17
	Improved	3 (60%)	0 (0%)	
	Unchanged	2 (40%)	4 (100%)	
Normal		*n* = 3	*n* = 5	1.00
	Maintained	2 (67%)	4 (80%)	
	Deteriorated	1 (33%)	1 (20%)	

Values are presented as number (percentage) of participants. *P* values were calculated based on Fisher's exact test. One participant was excluded from the water group because an incorrect taste identification during the baseline measurement prevented determination of the threshold. Reduced: participants with reduced taste sensitivity at baseline; Normal: those with normal taste sensitivity at baseline; Improved: those with reduced sensitivity at baseline and whose sensitivity improved to the normal range at 4 weeks; Unchanged: those with reduced sensitivity at baseline whose sensitivity remained in the reduced range; Maintained: those with normal sensitivity at baseline whose sensitivity remained in the normal range; Deteriorated: those with normal sensitivity at baseline whose sensitivity declined to the reduced range at 4 weeks.

### 3.3 Secondary outcomes

Figure 2 shows the changes in food preferences over 4 weeks. No statistically significant between-group differences were observed in food preference changes after the 4-week intervention. In exploratory within-group analyses, preference for the non-sweet fat subgroup and sweet carbohydrate foods decreased in the dashi group (nominal *p* = 0.02 for both), whereas the nominal within-group *p*-value for total fat preference was 0.05. The corresponding adjusted between-group differences were −11 points (95% CI, −28 to 7; *p* = 0.22) for non-sweet fat preference and −8 points (95% CI, −22 to 7; *p* = 0.29) for sweet carbohydrate preference. No significant within-group changes were observed in the water group. Detailed food preference data are presented in Table 4.

**Figure 2:**
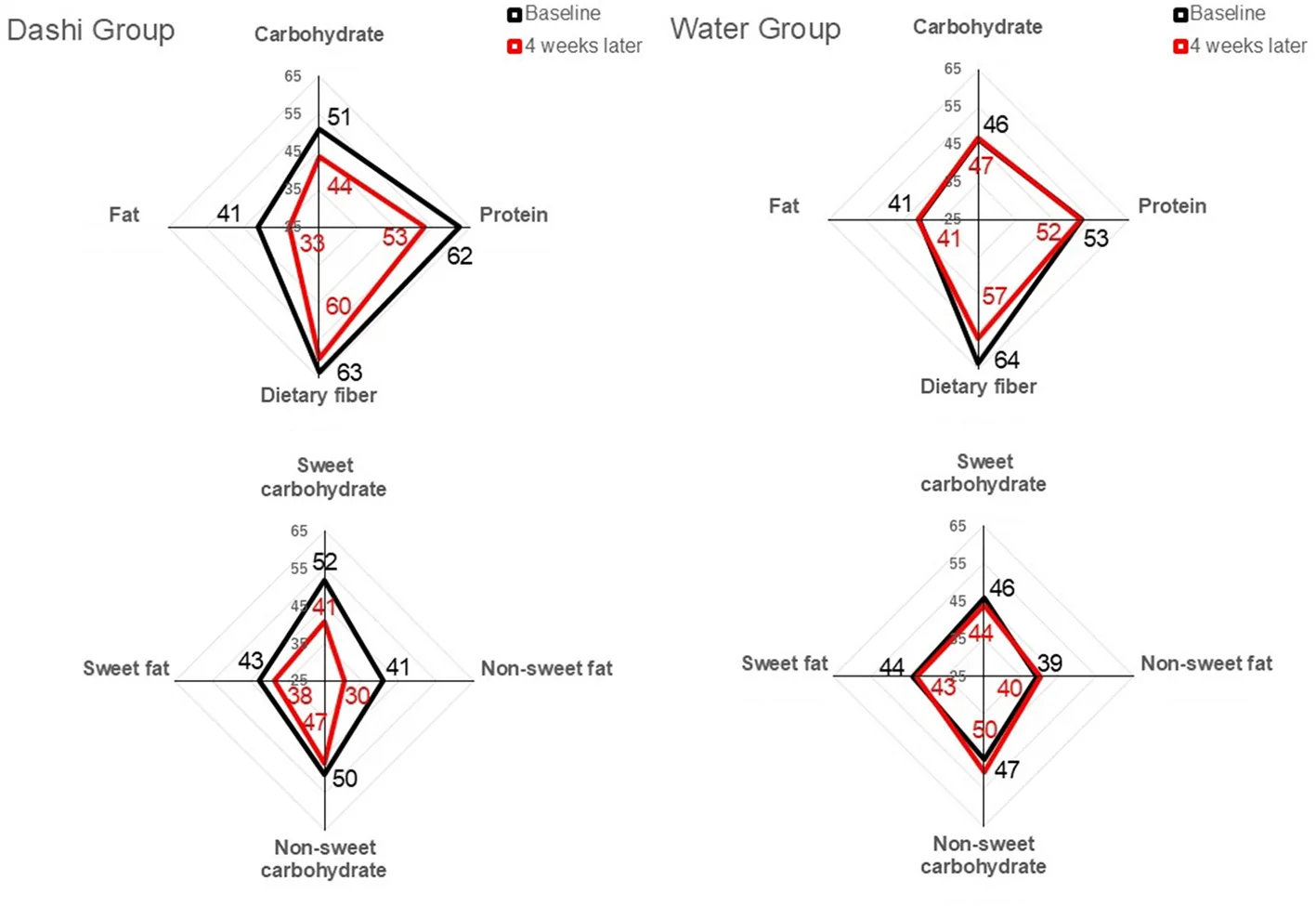
Changes in overall and subcategory food preferences in patients with type 2 diabetes after 4 weeks of dashi or water consumption. Radar charts illustrate changes in food preferences by nutrient group among patients with type 2 diabetes who consumed dashi **(left)** or water **(right)** before meals for the initial 4 weeks. Black lines represent baseline preferences, and red lines represent preferences after 4 weeks. Numerical labels indicate mean scores. Between-group differences, 95% confidence intervals, and *p*-values are presented in Table 4.

**Table 4:** Changes in food preferences in patients with type 2 diabetes after 4 weeks of dashi or water consumption.

	Dashi group (*n* = 8)			Water group (*n* = 10)			Intergroup	
Food preference category	Baseline	4 weeks	Within-group *p* value	Baseline	4 weeks	Within-group *p* value	Adjusted between-group difference (95% CI)	Adjusted *p* value
Carbohydrate	51 ±21	44 ± 20	0.17	46 ±13	47 ±16	0.94	−6 (−21, 8)	0.37
Sweet	52 ± 22	41 ± 21	0.02	46 ± 15	44 ± 18	0.69	−8 (−22, 7)	0.29
Non-sweet	50 ± 22	47 ± 19	0.64	47 ±13	50 ± 17	0.59	−5 (−21, 12)	0.55
Fat	41 ± 19	33 ± 17	0.05	41 ± 18	41 ± 19	0.96	−8 (−24, 7)	0.27
Sweet	43 ± 22	38 ± 27	0.41	44 ± 20	43 ± 20	0.82	−3 (−17, 10)	0.61
Non-sweet	41 ± 18	30 ± 14	0.02	39 ± 18	40 ± 22	0.93	−11 (−28, 7)	0.22
Protein	62 ± 29	53 ± 24	0.15	53 ± 16	52 ± 14	0.96	−5 (−19, 9)	0.43
Dietary fiber	63 ± 32	60 ± 28	0.35	64 ± 11	57 ± 17	0.23	3 (−11, 17)	0.66

Values are presented as mean ± standard deviation. Scores are expressed as the percentage of the maximum possible total score for each food preference category (0–100%). Adjusted between-group differences, corresponding 95% CIs, and *p*-values were estimated using ANCOVA. CI, confidence interval. Within-group *p*-values were calculated using paired *t*-tests, except for sweet carbohydrate foods preference in the dashi group, which was analyzed using the Wilcoxon signed-rank test.

Results for salty and umami taste sensitivity are presented in Supplementary Table 2. Among participants with reduced baseline taste sensitivity, improvement was observed in 1 of 5 participants for salty taste and 2 of 6 participants for umami taste in the dashi group, compared with 0 of 5 and 1 of 6 participants, respectively, in the water group. These findings should be interpreted as exploratory because the study was not powered to detect between-group differences.

No statistically significant differences were observed in biomarkers, body composition, or dietary intakes over the initial 4-week intervention period (Supplementary Tables 3 and 4). Descriptive outcomes at baseline, week 4, and week 8 are presented in Supplementary Tables 5 and 6.

## 4 Discussion

In this pilot randomized controlled trial, no statistically significant between-group differences were observed in the efficacy outcomes after 4 weeks of intervention. In exploratory within-group analyses, reductions in preference for non-sweet fatty foods and sweet carbohydrate foods were observed in the dashi group. A pattern of change in sweet taste sensitivity was also observed, particularly among participants with reduced sweet taste sensitivity at baseline. The mean within-group reductions in preference for non-sweet fatty foods and sweet carbohydrate foods were both approximately 11 points on the 0–100 JFPQ scale. However, the corresponding adjusted between-group differences were −11 points (95% CI, −28 to 7) and −8 points (−22 to 7) respectively, indicating considerable uncertainty regarding the magnitude and clinical relevance of the observed differences. Because a minimal clinically important difference for the JFPQ has not been established, an approximately 11-point within-group change cannot be interpreted as clinically meaningful. These findings should be considered exploratory and hypothesis-generating because of the pilot nature of the study and should not be interpreted as evidence of a treatment effect. To our knowledge, this is the first randomized study to explore the potential effects of a practical dashi-based intervention on food preferences and taste sensitivity in patients with type 2 diabetes.

These exploratory observations may be broadly consistent with previous experimental studies showing that MSG-containing broths can reduce subsequent intake of high-fat snacks or savory foods (24). Our study differs from prior work in two important respects: it evaluated a food-based dashi stimulus with synergistic umami components rather than MSG alone, and it focused on patients with type 2 diabetes, a population in whom altered sweet taste sensitivity and problematic food preferences may interfere with dietary self-management.

Several mechanisms proposed in previous studies may help explain the exploratory changes in food preferences observed in the present study. Taste signaling involves neurotransmitter-mediated communication within taste buds (25). Chronic exposure to high-fat and high-sugar diets has been shown to alter central reward pathways, including dopaminergic signaling, and to induce neuroendocrine dysregulation associated with increased preference for energy-dense foods (26–28). In addition, a previous randomized trial reported that repeated consumption of MSG-supplemented broth reduced both the desire for and intake of savory foods, suggesting that repeated umami exposure may modify subsequent food-related responses (16). In the present study, exploratory within-group reductions were observed in the dashi group in preferences for non-sweet fatty food and sweet carbohydrate foods, whereas the adjusted between-group differences were not statistically significant. These within-group observations do not establish a treatment effect. Although the underlying mechanisms were not directly investigated in the present study, the observed within-group reductions raise the hypothesis that dashi consumption could influence food preferences through sensory and reward-related pathways. Further studies are needed to clarify whether taste-based interventions can influence these pathways and contribute to long-term changes in dietary behavior.

Changes in sweet taste sensitivity may also be relevant to the observed findings. In this exploratory analysis, 3 of 5 patients (60%) with reduced sweet taste sensitivity at baseline in the dashi group and 0 of 4 (0%) in the water group improved to normal sensitivity (*p* = 0.17); given the very small numbers, this finding should not be interpreted as evidence of a subgroup effect. A previous study reported that stimulation of the tongue with MSG increased mRNA expression of taste receptors such as T1R1 and T1R3, the latter of which is also involved in sweet taste perception (29). However, taste-receptor expression was not measured in the present study, and whether this mechanism contributed to the exploratory findings warrants further investigation.

Although exploratory within-group changes in food preference scores were observed over 4 weeks, these changes were not accompanied by measurable improvements in dietary intake, biomarkers, or body composition. Therefore, these findings should not be interpreted as evidence of clinical benefit. The high completion and adherence rates support the feasibility of pre-meal dashi consumption as a sensory-based nutritional strategy, but do not provide evidence of metabolic efficacy in patients with type 2 diabetes. Longer intervention periods and larger-scale randomized controlled trials are required to determine whether these behavioral changes translate into clinically meaningful metabolic benefits. If confirmed in larger trials, such an approach may complement conventional dietary strategies by facilitating shifts in food preferences rather than relying solely on conscious dietary restraint.

This study has several strengths. It employed a randomized controlled design, a simple and feasible pre-meal intervention with high adherence, and repeated assessments of both food preference and taste sensitivity. The use of the JFPQ also allowed nutrient-based characterization of food preference in a Japanese population (8).

However, several limitations should be acknowledged. First, only 20 participants were randomized, despite the larger planned sample size, because recruitment was slower than anticipated during the study period. This smaller-than-planned sample size reduced statistical power, increased the risk of type II error, and limited the precision of the estimated effects. Therefore, this trial did not demonstrate superiority of dashi over hot water, and the efficacy findings remain inconclusive. The observed within-group changes should be interpreted as exploratory and hypothesis-generating. The small sample size and single-center design in Japanese patients with type 2 diabetes also limit the generalizability of the findings to other populations and healthcare settings. In addition, two of 10 participants in the dashi group did not complete the week-4 assessment, whereas all 10 participants in the water group completed it; because the efficacy analyses were complete-case analyses, this differential attrition may have influenced the estimates. Second, multiple non-primary and exploratory outcomes were evaluated without adjustment for multiple comparisons. Given the exploratory nature of this pilot study, multiplicity adjustments were not applied to these outcomes, and the corresponding *p*-values should be interpreted as unadjusted nominal *p*-values rather than as confirmatory evidence of statistical significance. The possibility of false-positive findings cannot be excluded. Accordingly, the two nominal within-group *p*-values of 0.02 should be interpreted particularly cautiously and may represent chance findings. Formal responsiveness and a minimal clinically important difference for the JFPQ have not been established, limiting interpretation of short-term score changes. Third, the trial was open-label, and expectation effects related to group assignment cannot be excluded. This is particularly relevant for subjective outcomes such as self-reported food preferences, which may be influenced by reporting bias or behavioral modification due to participants' awareness of the intervention. Furthermore, taste sensitivity was evaluated based on participants' self-reported perception of taste identification and therefore may also be susceptible to expectation-related bias. The baseline and week-4 taste assessments were generally performed by the same investigator, who was aware of group assignment; therefore, assessor-related measurement bias cannot be excluded. In addition, dietary intake was assessed using a self-reported food frequency questionnaire and may therefore be subject to reporting bias. Fourth, the use of hot water as a control condition represents a methodological limitation of this study. While it provided a simple and feasible comparator, it does not match dashi in terms of sensory characteristics, including aroma, flavor, and umami perception. It also does not control for intervention-related expectations. Therefore, it is difficult to determine whether the observed effects were attributable specifically to umami stimulation or to other sensory differences between the interventions. Thus, the study cannot isolate an umami-specific effect, and the findings should be interpreted as relating to dashi consumption as a whole. Future studies should consider the use of a flavor-matched control. Fifth, the observed improvement in taste sensitivity at week 4 may partly reflect increased familiarity with the testing procedure. Because taste solutions were presented in ascending concentrations, repeated testing may also have introduced a learning effect. In addition, the relatively short 4-week intervention period limited the assessment of the persistence of the exploratory changes. Larger and longer-term studies are needed to confirm these findings and evaluate their persistence. Sixth, the second intervention period was conducted without a washout interval. Therefore, carryover and period effects could not be excluded, and second-period findings should be interpreted descriptively. The decision to summarize second-period findings descriptively was not prespecified and was adopted after the potential carryover issue was recognized. Finally, although the role of the sponsor was clearly defined and statistical analyses were performed independently by the academic investigators, replication by independent research groups is needed to establish whether pre-meal dashi consumption has clinical utility.

In conclusion, this pilot randomized controlled trial suggests that pre-meal dashi consumption was feasible, but superiority over hot water was not demonstrated. Exploratory within-group changes in preference for non-sweet fatty foods and sweet carbohydrate foods, and in sweet taste sensitivity, should be considered hypothesis-generating and should not be interpreted as evidence of a treatment effect. Further large-scale, adequately powered and independently conducted trials are needed to determine whether these exploratory behavioral and sensory changes translate into sustained improvements in dietary behavior and metabolic outcomes.

## Data Availability

The datasets used during the current study are available from the corresponding author on reasonable request.

## References

[1] Deslippe AL Soanes A Bouchaud CC Beckenstein H Slim M Plourde H . Barriers and facilitators to diet, physical activity and lifestyle behavior intervention adherence: a qualitative systematic review of the literature. Int J Behav Nutr Phys Act. (2023) 20:14. doi: 10.1186/s12966-023-01424-2 PMC9925368 36782207

[2] Drewnowski A Kurth C Holden-Wiltse J Saari J. Food preferences in human obesity: carbohydrates versus fats. Appetite. (1992) 18:207–21. doi: 10.1016/0195-6663(92)90198-F 1510463

[3] Qiang Y Lu X Zhang Y. Association between dietary patterns and glycemic control in type ii diabetes mellitus patients. Aten Primaria. (2025) 57:103075. doi: 10.1016/j.aprim.2024.103075 39288729 PMC11421999

[4] Gondivkar SM Indurkar A Degwekar S Bhowate R. Evaluation of gustatory function in patients with diabetes mellitus type 2. Oral Surg Oral Med Oral Pathol Oral Radiol Endod. (2009) 108:876–80. doi: 10.1016/j.tripleo.2009.08.015 19913725

[5] Yu JH Shin MS Lee JR Choi JH Koh EH Lee WJ . Decreased sucrose preference in patients with type 2 diabetes mellitus. Diabetes Res Clin Pract. (2014) 104:214–9. doi: 10.1016/j.diabres.2014.02.007 24629412

[6] Pugnaloni S Alia S Mancini M Santoro V Di Paolo A Rabini RA . A study on the relationship between type 2 diabetes and taste function in patients with good glycemic control. Nutrients. (2020) 12:1112. doi: 10.3390/nu12041112 32316314 PMC7230282

[7] Tsujimoto T Imai K Kanda S Kakei M Kajio H Sugiyama T. Sweet taste disorder and vascular complications in patients with abnormal glucose tolerance. Int J Cardiol. (2016) 221:637–41. doi: 10.1016/j.ijcard.2016.07.062 27423082

[8] Nagai N Fujishima Y Tokuzawa C Takayanagi S Yamamoto M Hara T . Food preference assessed by the newly developed nutrition-based japan food preference questionnaire and its association with dietary intake in abdominal-obese subjects. Nutrients. (2024) 16:4252. doi: 10.3390/nu16234252 39683643 PMC11644194

[9] Salvia MG Quatromoni PA. Behavioral approaches to nutrition and eating patterns for managing type 2 diabetes: a review. Am J Med Open. (2023) 9:100034. doi: 10.1016/j.ajmo.2023.100034 39035058 PMC11256231

[10] Drewnowski A. Taste preferences and food intake. Annu Rev Nutr. (1997) 17:237–53. doi: 10.1146/annurev.nutr.17.1.237 9240927

[11] Yamaguchi S Yoshikawa T Ikeda S Ninomiya T. The synergistic taste effect of monosodium glutamate and disodium 5'-guanylate. Nippon Nogeikagaku Kaishi. (1968) 42:378–81. doi: 10.1271/nogeikagaku1924.42.6_378

[12] Ninomiya K. Science of umami taste: adaptation to gastronomic culture. Flavour. (2015) 4:13. doi: 10.1186/2044-7248-4-13

[13] Tsurugizawa T Kondoh T Torii K. Forebrain activation induced by postoral nutritive substances in rats. Neuroreport. (2008) 19:1111–5. doi: 10.1097/WNR.0b013e328307c414 18596610

[14] Masic U Yeomans MR. Umami flavor enhances appetite but also increases satiety. Am J Clin Nutr. (2014) 100:532–8. doi: 10.3945/ajcn.113.080929 24944058

[15] Miyaki T Imada T Hao SS Kimura E. Monosodium L-glutamate in soup reduces subsequent energy intake from high-fat savoury food in overweight and obese women. Br J Nutr. (2016) 115:176–84. doi: 10.1017/S0007114515004031 26455957

[16] Noel CA Finlayson G Dando R. Prolonged exposure to monosodium glutamate in healthy young adults decreases perceived umami taste and diminishes appetite for savory foods. J Nutr. (2018) 148:980–8. doi: 10.1093/jn/nxy055 29796671

[17] Beppu K Shono H Kawakami A Takashi T Watanabe S Yoshida A . Dietary supplementation with monosodium glutamate with dietary balance such as protein, salt and sugar intake with increasing t1r3 taste receptor gene expression in healthy females. J Med Invest. (2021) 68:315–20. doi: 10.2152/jmi.68.315 34759151

[18] Hosaka H Kusano M Zai H Kawada A Kuribayashi S Shimoyama Y . Monosodium glutamate stimulates secretion of glucagon-like peptide-1 and reduces postprandial glucose after a lipid-containing meal. Aliment Pharmacol Ther. (2012) 36:895–903. doi: 10.1111/apt.12050 22978669

[19] Anderson GH Fabek H Akilen R Chatterjee D Kubant R. Acute effects of monosodium glutamate addition to whey protein on appetite, food intake, blood glucose, insulin and gut hormones in healthy young men. Appetite. (2018) 120:92–9. doi: 10.1016/j.appet.2017.08.020 28843973

[20] Shono H Tsutsumi R Beppu K Matsushima R Watanabe S Fujimoto C . Dietary supplementation with monosodium glutamate suppresses chemotherapy-induced downregulation of the t1r3 taste receptor subunit in head and neck cancer patients. Nutrients. (2021) 13:2921. doi: 10.3390/nu13092921 PMC8469378 34578798

[21] Hara T Fujishima Y Kimura Y Fujii K Kawachi Y Nagao H . Effects of semaglutide, a glp-1 receptor agonist, on food preferences in japanese subjects with type 2 diabetes and visceral fat accumulation. Endocr J. (2026) 73:607–19. doi: 10.1507/endocrj.EJ25-0402 41605684 PMC13195457

[22] Kawakami I Ganeko N Ikegami Y Yunoue Y Matsuzoe N Kitano N. Taste sensitivity and preference for dashi in female university students. Jpn J Nutr Diet. (2011) 69:10–9. doi: 10.5264/eiyogakuzashi.69.10

[23] Huang X Guo Y Wei Y. [Reliability of whole-mouth taste test in assessment of gustatory function in healthy adults]. Lin Chuang Er Bi Yan Hou Tou Jing Wai Ke Za Zhi. (2021) 35:698–701. doi: 10.13201/j.issn.2096-7993.2021.08.006 PMC10127804 34304529

[24] Imada T Hao SS Torii K Kimura E. Supplementing chicken broth with monosodium glutamate reduces energy intake from high fat and sweet snacks in middle-aged healthy women. Appetite. (2014) 79:158–65. doi: 10.1016/j.appet.2014.04.011 24768895

[25] Roper SD Chaudhari N. Processing umami and other tastes in mammalian taste buds. Ann N Y Acad Sci. (2009) 1170:60–5. doi: 10.1111/j.1749-6632.2009.04107.x PMC3717262 19686109

[26] Reyes TM. High-fat diet alters the dopamine and opioid systems: effects across development. Int J Obes Suppl. (2012) 2:S25–8. doi: 10.1038/ijosup.2012.18 PMC4850606 27152150

[27] Atar A. Neurobiological consequences of high-fat high-sugar diets on the mesocorticolimbic system: a narrative review. Curr Nutr Rep. (2026) 15:6. doi: 10.1007/s13668-026-00729-5 41543783 PMC12811340

[28] Roca M Íbias J Mohamedi-Ahmed Y Ruiz-Gayo M Ambrosio E Sanz-Martos AB . Saturated and unsaturated high-fat diets induce addictive-like behavior in an animal model of operant self-administration. Prog Neuropsychopharmacol Biol Psychiatry. (2025) 137:111295. doi: 10.1016/j.pnpbp.2025.111295 39988255

[29] Shoji N Kaneta N Satoh-Kuriwada S Tsuchiya M Hashimoto N Uneyama H . Expression of umami-taste-related genes in the tongue: a pilot study for genetic taste diagnosis. Oral Dis. (2015) 21:801–6. doi: 10.1111/odi.12350 25990784

