# Supplementary material for: Effects of pre-meal dashi consumption on food preferences and sweet taste sensitivity in patients with type 2 diabetes: a pilot randomized controlled trial
Source: Front Nutr. 2026 Oct 28;13:1903008. doi: 10.3389/fnut.2026.1903008 (PMC13754174; doi:10.3389/fnut.2026.1903008)
Supplement: Supplementary file 1 [file Table_1.docx]

**Supplementary Materials**

**Table of contents**

**Supplementary Table 1. Taste solution concentrations
Supplementary Table 2. Changes in salty and umami taste sensitivity according to baseline sensitivity
Supplementary Table 3. Changes in biomarkers and body composition before and after 4 weeks
Supplementary Table 4. Changes in dietary intakes before and after 4 weeks
Supplementary Table 5. Taste thresholds at baseline, week 4, and week 8
Supplementary Table 6. Japan Food Preference Questionnaire scores at baseline, week 4, and week 8**

**Supplementary Table 1. Taste solution concentrations**

| **Concentration level** | **Sweetness (sucrose, g/L)** | **Salty (sodium chloride, g/L)** | **Umami (MSG, g/L)** |
| --- | --- | --- | --- |
| 1 | 1.25 | 0.15 | 0.2 |
| 2 | 2.5 | 0.3 | 0.4 |
| 3 | 5 | 0.6 | 0.8 |
| 4 | 10 | 1.2 | 1.6 |
| 5 | 20 | 2.4 | 3.2 |
| 6 | 40 | 4.8 | 6.4 |
| 7 | 80 | 9.6 | 12.8 |
| 8 | 120 | 14.4 | 19.2 |

MSG; monosodium glutamate.

**Supplementary Table 2. Changes in salty and umami taste sensitivity according to baseline sensitivity**

| **Taste** | **Baseline** | **Outcome** | **Dashi group（n = 8)** | **Water group (n = 10)** | **p-value** |
| --- | --- | --- | --- | --- | --- |
| Salty | Reduced |  | n=5 | n=5 | 1.00 |
|  |  | Improved | 1 (20%) | 0 (0%) |  |
|  |  | Unchanged | 4 (80%) | 5 (100%) |  |
|  | Normal |  | n=3 | n=5 | 0.11 |
|  |  | Maintained | 1 (33%) | 5 (100%) |  |
|  |  | Deteriorated | 2 (67%) | 0 (0%) |  |
| Umami | Reduced |  | n=6 | n=6 | 1.00 |
|  |  | Improved | 2 (33%) | 1 (17%) |  |
|  |  | Unchanged | 4 (67%) | 5 (83%) |  |
|  | Normal |  | n=2 | n=4 | 1.00 |
|  |  | Maintained | 1 (50%) | 3 (75%) |  |
|  |  | Deteriorated | 1 (50%) | 1 (25%) |  |

Values are number (percentage) of participants for categorical variables. P value is calculated based on Fisher’s exact test.

Reduced, participants with reduced taste sensitivity at baseline. Normal, participants with normal taste sensitivity at baseline.

**Supplementary Table 3. Changes in biomarkers and body composition before and after 4 weeks**

| **Measure** | **Dashi group (n = 8)** | | | **Water group (n = 10)** | | | **Intergroup** | |
| --- | --- | --- | --- | --- | --- | --- | --- | --- |
|  | Baseline | 4 weeks | Within-group p | Baseline | 4 weeks | Within-group p | Adjusted between-group difference  (95% CI) | Adjusted p value |
| BMI (kg/m^2^) | 25.5 ± 3.2 | 25.5 ± 3.1 | 0.72 | 24.8 ± 2.6 | 24.8 ± 2.6 | 1.00 | 0 (-0.3, 0.4) | 0.74 |
| TG (mmol/L) | 1.51 ± 0.83 | 1.42 ± 0.93 | 0.56 | 1.01 ± 0.27 | 1.05 ± 0.23 | 0.69 | -0.08 (-0.50, 0.33) | 0.68 |
| LDL-C (mmol/L) | 2.67 ± 0.77 | 2.67 ± 0.74 | 0.93 | 2.52 ± 0.51 | 2.46 ± 0.54 | 0.56 | 0.07 (-0.19, 0.34) | 0.56 |
| HbA1c (NGSP, %) | 7.6 ± 0.7 | 7.6 ± 0.8 | 1.00 | 7.1 ± 0.8 | 7.2 ± 0.8 | 0.40 | 0 (-0.3, 0.4) | 0.86 |
| eGFR (mL/min/1.73 m²) | 70 ± 34 | 71 ± 36 | 0.60 | 70 ± 29 | 72 ± 29 | 0.14 | -1 (-5, 4) | 0.79 |
| AST (U/L) | 18 ± 4 | 19 ± 4 | 0.10 | 18 ± 4 | 17 ± 4 | 0.71 | 2 (0, 4) | 0.12 |
| ALT (U/L) | 15 ± 5 | 16 ± 6 | 0.33 | 18 ± 8 | 16 ± 6 | 0.23 | 2 (-1, 5) | 0.25 |
| Fat mass (kg) | 21.1 ± 8.3 | 21.4 ± 8.2 | 0.41 | 19.1 ± 6.0 | 19.2 ± 5.5 | 0.65 | 0.2 (-0.8, 1.2) | 0.73 |
| Lean mass (kg) | 41.4 ± 4.3 | 41.3 ± 4.6 | 0.80 | 43.6 ± 10.2 | 43.5 ± 10.1 | 0.57 | 0 (-0.8, 0.7) | 0.98 |

Values are mean ± standard deviation. Within-group analyses are exploratory and presented for descriptive purposes. Adjusted between-group differences, corresponding 95% CIs, and p-values were estimated using ANCOVA. BMI, Body Mass Index. TG, triglyceride; LDL-C, low-density lipoprotein cholesterol; NGSP, National Glycohemoglobin Standardization Program; AST, aspartate aminotransferase; ALT, alanine aminotransferase; CI, confidence interval. Within-group p-values were calculated using paired t-tests, except for HbA1c and AST in the dashi group, which were analyzed using the Wilcoxon signed-rank test.

**Supplementary Table 4. Changes in dietary intakes before and after 4 weeks**

| **Nutrient** | **Dashi group (n = 8)** | | | **Water group (n = 10)** | | | **Intergroup** | |
| --- | --- | --- | --- | --- | --- | --- | --- | --- |
|  | Baseline | 4 weeks | Within-group p | Baseline | 4 weeks | Within-group p | Adjusted between-group difference  (95% CI) | Adjusted p value |
| Energy (kcal) | 2024 ± 223 | 2015 ± 185 | 0.87 | 2112 ± 219 | 2092 ± 224 | 0.70 | -15 (-161, 132) | 0.84 |
| Carbohydrate (g) | 265 ± 45 | 259 ± 24 | 1.00 | 284 ± 45 | 277 ± 41 | 1.00 | -8 (-37, 21) | 0.57 |
| Fiber (g) | 18 ± 1 | 18 ± 1 | 0.11 | 19 ± 2 | 19 ± 3 | 0.41 | 0 (-2, 2) | 0.86 |
| Protein (g) | 82 ± 7 | 81 ± 8 | 0.73 | 85 ± 7 | 84 ± 8 | 0.58 | 0 (-6, 5) | 0.86 |
| Lipid (g) | 62 ± 3 | 63 ± 3 | 0.74 | 64 ± 4 | 64 ± 5 | 0.90 | -1 (-5,4) | 0.69 |
| Saturated lipid (g) | 18 ± 1 | 19 ± 1 | 0.39 | 19 ± 1 | 19 ± 2 | 1.00 | 0 (-2,2) | 0.90 |
| Salt (g) | 11 ± 1 | 11 ± 1 | 0.86 | 11 ± 2 | 11 ± 2 | 0.80 | 0 (-1, 2) | 0.60 |

Values are mean ± standard deviation. Within-group analyses are exploratory and presented for descriptive purposes. Adjusted between-group differences, corresponding 95% CIs, and p-values were estimated using ANCOVA. FFQ, Food Frequency Questionnaire; CI, confidence interval; SD, standard deviation. Within-group p-values were calculated using paired t-tests, except for the carbohydrate intake in both the dashi and water groups, the fiber intake in the water group, the saturated lipid intake in the water group, and the salt intake in the water group, for which the Wilcoxon signed-rank test was used.

**Supplementary Table 5. Taste thresholds at baseline, week 4, and week 8**

| **Taste threshold** | **Group** | **N** | **Baseline** | **Week 4** | **Week 8** |
| --- | --- | --- | --- | --- | --- |
| Sweet taste threshold (g/L) | Dashi→Water | 8 | 30.0 ± 38.1 | 13.1 ± 5.9 | 16.9 ± 11.0 |
|  | Water→Dashi | 9 | 25.6 ± 24.0 | 17.8 ± 9.7 | 14.4 ± 5.3 |
| Salty taste threshold (g/L) | Dashi→Water | 8 | 2.0 ± 1.5 | 2.1 ± 1.4 | 1.8 ± 0.9 |
|  | Water→Dashi | 10 | 1.1 ± 0.8 | 1.1 ± 0.8 | 1.3 ± 1.3 |
| Umami taste threshold (g/L) | Dashi→Water | 8 | 2.3 ± 2.0 | 1.6 ± 1.1 | 5.0 ± 7.0 |
|  | Water→Dashi | 10 | 2.7 ± 3.7 | 2.1 ± 1.8 | 3.0 ± 5.7 |

Data are presented as mean ± standard deviation.

**Supplementary Table 6. Japan Food Preference Questionnaire scores at baseline, week 4, and week 8**

| **Food Preference category** | **Group** | **N** | **Baseline** | **Week 4** | **Week 8** |
| --- | --- | --- | --- | --- | --- |
| Carbohydrate | Dashi→Water | 8 | 51 ± 21 | 44 ± 20 | 43 ± 18 |
|  | Water→Dashi | 10 | 46 ± 13 | 47 ± 16 | 51 ± 13 |
| Fat | Dashi→Water | 8 | 41 ± 19 | 33 ± 17 | 32 ± 19 |
|  | Water→Dashi | 10 | 41 ± 18 | 41 ± 19 | 39 ± 19 |
| Protein | Dashi→Water | 8 | 62 ± 29 | 53 ± 24 | 53 ± 27 |
|  | Water→Dashi | 10 | 53 ± 16 | 52 ± 14 | 56 ± 17 |
| Dietary fiber | Dashi→Water | 8 | 63 ± 32 | 60 ± 28 | 52 ± 25 |
|  | Water→Dashi | 10 | 64 ± 11 | 57 ± 17 | 59 ± 17 |

Data are presented as mean ± standard deviation. Scores are expressed as the percentage of the maximum possible total score for each food preference category (0–100%).
